# Congregation of Medical Men in Atlanta

**Published:** 1879-04

**Authors:** 


### CONGREGATION OF MEDICAL MEN IN ATLANTA.

The first week of May will afford Atlanta the most important and numerous association of medical men that ever assembled at one time in America. Important, not only on account of the number of learned and distinguished members of the profession who will be present, but more particularly on account of the subjects to be discussed, and the objects to be accomplished by the meetings.

The National Board of Health, recently appointed, will hold its first regular meeting in Atlanta on the first of May; the Convention of American Medical Colleges will convene in the Senate Chamber of the Capitol in Atlanta, on the second of May; the American Medical College Association will meet in the same room on Saturday, the third day of May; and the American Medical Association will meet in the Representative Hall of the same building on Tuesday, the sixth day of May.

The three meetings preceding that of the American Medical Association, are intended to discuss and decide upon very grave questions. Questions, which involve not only the well being of the medical profession, but also that of the public generally. The public interest depends in no small degree upon the conclusions arrived at by the National Board of Health, and professional progress and success are to some extent in their hands. Members of this Board had an organization meeting in Washington recently, and decided upon the provisions of a bill for the consideration of Congress, to promote the sanitary condition of the country. Their investigations seem to be more immediately connected with the introduction and spread of contagious and infectious diseases. Their recommendations to Congress it appears, were sent to certain Senate Committees, but of the particulars in these suggestions we are not informed. Whether or not a national quarantine law is urged, and if so, against what particular contagions and infections, we have as yet had no means of information. We know that toward the conclusion of the last Congress, a stringent national quarantine bill passed the Senate of the United States, but failed in the House. This bill, as we understood it, was intended to guard our coasts against the introduction of yellow fever, and secular papers are insisting on action to this effect being taken before the adjournment of the called session. Members of Congress, however, begin to see that the question upon which quarantining against yellow fever must depend, has at any rate, two sides to it, and there is some doubt whether or not the subject will receive serious consideration from Congress at present.

The Convention of Medical Colleges, consisting of two delegates from each accredited school of medicine, which meets on the second day of May, is expected to discuss and pass upon very serious questions connected with medical education. Chief among them is that touching the increase of the number of courses in order to admission for examination.

A journalist remarks, that it is easy to make the plan for an ideal medical college, but to fix upon details practicable and acceptable to all, is quite another matter. While one may conclude that the addition of another course to the ordinary terms of study is altogether sufficient to insure the preparation of pupils, another will differ from him in *toto* and insist upon some other test of qualification than that of the vote of a faculty, who have been the teachers of, and sympathizers with, an unfortunate pupil. Separate says he, the teaching and licensing power, so that the examination by strangers may work justice to all applicants, and protect the public against uneducated practitioners. Let no time of study, which sometimes only increases the teachers sympathy, be the test of preparation, but his thorough knowledge of the sciences, which is necessary to practice successfully the healing art, whether it has required two years or twenty to obtain it.

The Association of American Medical Colleges, which meets on Saturday, has called the above Convention of Colleges, and will doubtless, at their opening session, receive a report of the action had. The discussions in the Convention on various points will shape up matters more compactly for the decision of the College Association, and thereby lessen the time necessary for their consideration.

The American Medical Association, having been relieved from the consideration of college questions by the bodies specially designed for that duty, will no doubt add greatly to scientific advancement and practical medical knowledge. These bodies will receive a hearty welcome from the profession and our people generally, and we hope every means in our power, to make them comfortable and happy, will be afforded. Already are live, active members of the local profession exerting themselves to arrange matters so that the comfort and pleasure of the visiting members may be perfectly secured.

It appears that the municipal authorities would do well to exhibit their enterprise and munificence by giving a helping hand. In this way an effect may be obtained that would redound no little to the credit of Atlanta, by increasing her reputation abroad.

